# Antimicrobial photodynamic therapy for inactivation of biofilms formed by oral key pathogens

**DOI:** 10.3389/fmicb.2014.00405

**Published:** 2014-08-12

**Authors:** Fabian Cieplik, Laura Tabenski, Wolfgang Buchalla, Tim Maisch

**Affiliations:** ^1^Department of Operative Dentistry and Periodontology, University Medical Center RegensburgRegensburg, Germany; ^2^Department of Dermatology, University Medical Center RegensburgRegensburg, Germany

**Keywords:** photodynamic, aPDT, antimicrobial, biofilm, oral, antibiotic resistance

## Abstract

With increasing numbers of antibiotic-resistant pathogens all over the world there is a pressing need for strategies that are capable of inactivating biofilm-state pathogens with less potential of developing resistances in pathogens. Antimicrobial strategies of that kind are especially needed in dentistry in order to avoid the usage of antibiotics for treatment of periodontal, endodontic or mucosal topical infections caused by bacterial or yeast biofilms. One possible option could be the antimicrobial photodynamic therapy (aPDT), whereby the lethal effect of aPDT is based on the principle that visible light activates a photosensitizer (PS), leading to the formation of reactive oxygen species, e.g., singlet oxygen, which induce phototoxicity immediately during illumination. Many compounds have been described as potential PS for aPDT against bacterial and yeast biofilms so far, but conflicting results have been reported. Therefore, the aim of the present review is to outline the actual state of the art regarding the potential of aPDT for inactivation of biofilms formed *in vitro* with a main focus on those formed by oral key pathogens and structured regarding the distinct types of PS.

## Introduction

Infections caused by bacterial biofilms are an immediate problem for public health as—according to the National Institutes of Health (NIH)—biofilm-associated diseases can be accounted for 80% of all infections in humans (PA-07-288: Immunology of Biofilms). According to Rodney M. Donlan and J. William Costerton the current definition of the term “biofilm” is as follows:

*“A biofilm is a microbially derived sessile community characterized by cells that are irreversibly attached to a substratum or interface or to each other, are embedded in a matrix of extracellular polymeric substances that they have produced, and exhibit an altered phenotype* [as compared to planktonic cells] *with respect to growth rate and gene transcription”* (Donlan and Costerton, 2002)

It is well known that the properties of bacteria embedded in biofilms are very different from those of their planktonic counterparts. For example, Shani et al. revealed that the concentrations of amine fluoride and chlorhexidine, which were able to kill monospecies biofilms of *Streptococcus sobrinus*, were about 100 times greater than the concentrations that were necessary to eradicate planktonic cultures of the same organism (Shani et al., 2000). Likewise, Ceri et al. showed that for eradication of biofilms of *Escherichia coli*, *Pseudomonas aeruginosa*, and *Staphylococcus aureus* even up to 1000 times higher concentrations of a certain antibiotic were required for the antibiotic to be effective compared to planktonic cells (Ceri et al., 1999). The enhanced tolerance of microorganisms growing in biofilms against antimicrobials can be explained as follows:

Firstly, the biofilm matrix—also referred to as EPS (extracellular polymeric substance)—itself may slow drug-diffusion by its higher viscosity or can even act as a barrier (Mah and O'Toole, 2001; Stewart and Costerton, 2001). For example, EPS-molecules are able to react with antimicrobials via redox-processes, positively charged agents bind to negatively charged EPS-molecules and π −π-interactions of aromatic surfaces are possible, preventing penetration of the respective drug in deeper parts of the biofilm (Mah and O'Toole, 2001; Stewart and Costerton, 2001).

Secondly, gene expression is altered between sessile and planktonic cells. Shemesh et al. demonstrated by a comparative transcriptome analysis for *Streptococcus mutans* that approximately 12% of genes showed a significantly dissimilar expression pattern in sessile and planktonic cells (Shemesh et al., 2007). Welin and Svensäter showed that in comparison to planktonic cells protein expression is altered in matured 3 days old biofilms as well as during the initial stage of biofilm formation; hereby they found that the expression of proteins related to the carbohydrate catabolism was elevated during initial attachment, whereas in matured biofilms it was reduced (Svensäter et al., 2001; Welin et al., 2004). Likewise, Lo et al. showed for *Porphyromonas gingivalis*, a periodontal pathogen, that approximately 18% of its genome was expressed distinctly when the bacterium was grown as monospecies biofilm; hereby, mainly genes involved in DNA replication and energy production were downregulated whereas a number of genes encoding binding and transport proteins were upregulated (Lo et al., 2009).

Furthermore, development of a biofilm leads to an enormous genetic diversity of its cells, which provides “insurance” for the cells for better adaption to abrupt alteration of environment conditions (Kolter and Greenberg, 2006). Boles et al. showed for *Pseudomonas aeruginosa* that biofilm-grown cells had varying colony morphologies when plated on agar, whereas planktonic-grown cells had not; analyzing these biofilm-grown variants revealed that they differed notably in their biofilm building and detachment properties, susceptibility to oxidative stress and other properties (Boles et al., 2004). Likewise, so-called persister cells are being formed, when bacteria grow as a biofilm. These are in a dormant, non-dividing state and provide tolerance toward antimicrobial agents. This tolerance might function by preventing target corruption by an antimicrobial due to blocking of the target (Lewis, 2007). In contrast, resistance means target modification by mutation or enzymatic changes, target substitution, destruction or modification of the antibiotic, emergence of efflux pumps or restricted permeability for antibiotics through cell walls (Lewis, 2007).

To date, resistances of bacteria against antibiotics (Rossolini and Mantengoli, 2008) and antimicrobials like chlorhexidine (CHX) (Yamamoto et al., 1988) and triclosan (Yazdankhah et al., 2006) are arising and there is a pressing need for development of new antimicrobial approaches to fight biofilm infections. In January 2009, Cesar A. Arias and Barbara E. Murray published an article in the *New England Journal of Medicine*, where they warned that bacteria might even become winners of evolution since several strains have already adapted toward antibiotic and antimicrobial treatment that they become more and more resistant to conventional therapies; thus, they announced that this will be a “clinical super-challenge in the 21st century” fighting the spread of resistance (Arias and Murray, 2009).

With respect to the situation in the oral cavity, dentists also are often faced with the situation that they have to combat antibiotic-resistant bacteria in periodontal (Rams et al., 2014) or endodontic (Al-Ahmad et al., 2014) infections. For this reason,—in addition to promoting an adequate and rationalized use of antimicrobials (Leung et al., 2011)—there is immediate and continual research in human and dental medicine for alternative methods with less potential of development of resistances in pathogens. Multi-target processes are needed in contrast to those of antibiotics, which act very specific toward one explicit target according the so-called key-hole principle (Alves et al., 2014).

**Figure 1:**
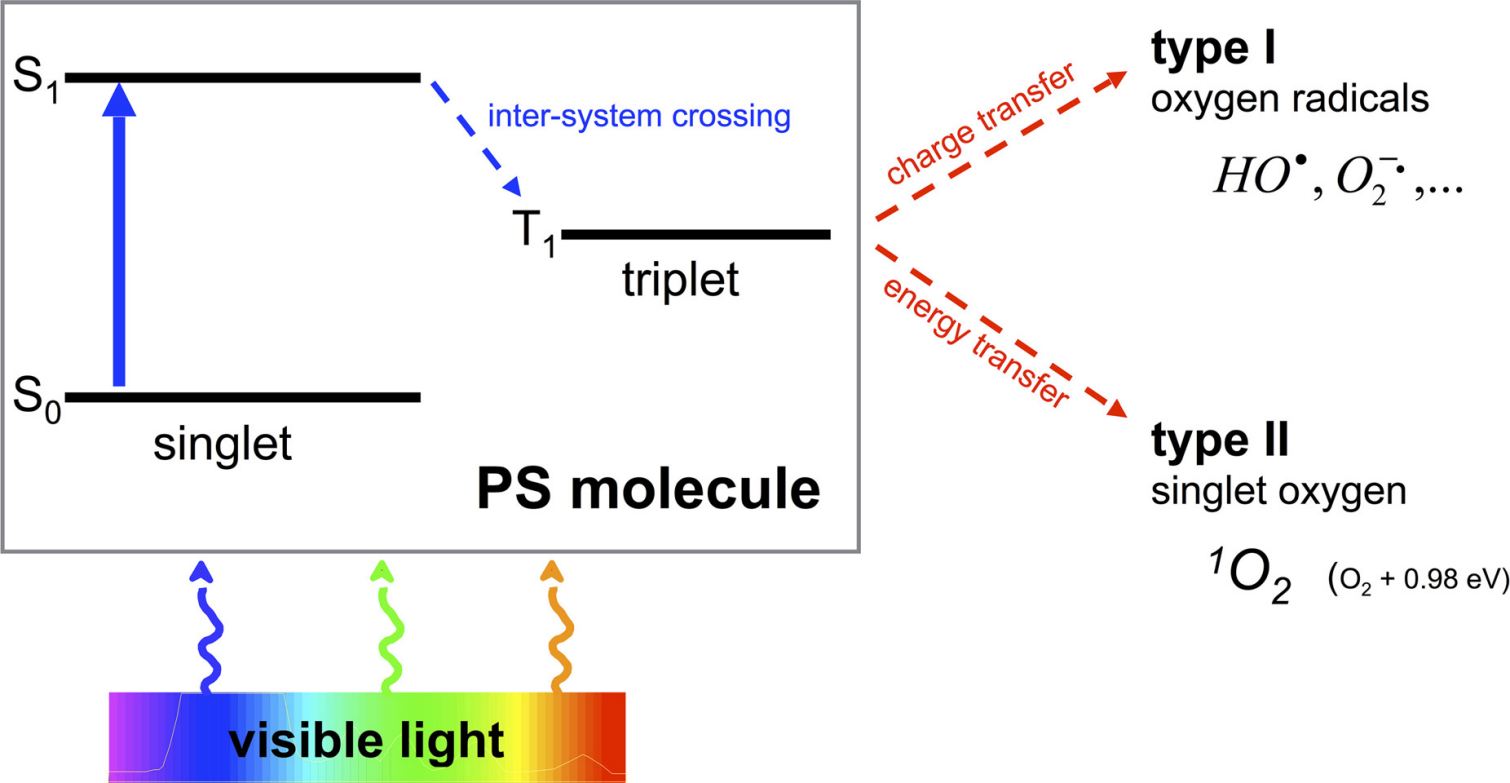
**Type I and type II processes of aPDT**. Visible light of an appropriate wavelength is absorbed by the PS molecule by what the PS changes from its initial ground state S_0_ to an energetically excited state S_1_. Thereupon the PS is able to transition within the molecule from its singlet to its triplet state T_1_ (inter-system crossing). This T_1_ state is long-living compared to S_1_ so that charge (type I) or energy (type II) can be transferred to surrounding molecules such as oxygen with emergence of oxygen radicals (type I) or singlet oxygen (type II).

One of the most promising approaches to overcome the aforementioned shortcomings is the antimicrobial Photodynamic Therapy (aPDT). Its antimicrobial effect is based on an oxidative burst upon illumination and relies on damage to cellular structures and biomolecules, therefore being an unspecific mechanism (Maisch et al., 2011). aPDT needs the presence of three components, (I) a *per se* non-toxic dye, the so-called photosensitizer (PS), (II) visible light of an appropriate wavelength, and (III) molecular oxygen. The absorption of light by the PS leads to a transition to its triplet state, whereupon there are two mechanisms of reaction to let the PS regain its ground state: In type I mechanism, charge is transferred to a substrate or to molecular oxygen generating reactive oxygen species like hydrogen peroxide and oxygen radicals like superoxide ions or free hydroxyl radicals. In type II mechanism, energy only—not charge—is transferred directly to molecular oxygen, whereby the highly reactive singlet oxygen (^1^O_2_) originates (Wainwright, 1998; Schweitzer and Schmidt, 2003) (see Figure 1). Hereby, the singlet oxygen quantum yield Φ_Δ_ describes the fraction of type II mechanism (Maisch et al., 2007).

In general, a PS used for aPDT should show the following features in order to have a pronounced antimicrobial efficacy and low toxicity toward mammalian cells:

- High ^1^O_2_ quantum yield Φ_Δ_ (Maisch et al., 2007).
- High binding affinity for microorganisms (positively charged PS for good adherence to negatively charged bacterial cell walls) (Alves et al., 2009).
- Low binding affinity for mammalian cells (Soukos and Goodson, 2011).
- Low chemical toxicity and mutagenicity (Soukos and Goodson, 2011).

The efficacy of many PS has already been evaluated for inactivation of biofilms grown *in vitro*: mainly there were used phenothiazinium dyes, such as Methylene Blue and Toluidine Blue, tetrapyrrolic macrocycles like porphyrins (e.g., TMPyP) or xanthene dyes like Erythrosine and Rose Bengal (RB). In addition, further chemical classes, e.g., functionalized fullerenes (Mizuno et al., 2011) or curcumin (Araújo et al., 2012, 2014) were presented. More recently a dye named SAPYR was introduced, which is based on a perinaphthenone structure (Cieplik et al., 2013b).

The aim of the present review is to outline the actual state of research concerning the effect of the photodynamic process for inactivation of bacterial and yeast biofilms *in vitro* with a main focus on aPDT against biofilms grown from oral key pathogens.

## aPDT against biofilms *in vitro*

In the following, only publications were included that investigated the efficacy of aPDT against bacterial and yeast biofilms grown *in vitro*. For ensuring better comparability, only studies were included, where the effect of aPDT was examined by performing colony forming unit (CFU) assays. CFU assay data is essential for evaluating a new antimicrobial approach, as the American Society of Microbiology (ASM) stated in 2010 that every new approach has to prove an efficacy of 3 log_10_ reduction of CFU before being able to use the terms “antimicrobial” or “antibacterial.”

### Phenothiazinium derivatives

The first phenothiazinium dyes were synthetized by the end of the 19th century in Germany in the context of a booming German textile industry. Hereby, Alizarin, which was developed in 1868 by Carl Graebe and Carl Liebermann, worked as some kind of precursor, when further research in the purpose of its manufacture and commercialization led to the development of new dyes (López-Muñoz et al., 2005). One of these new dyes was so-called “Methylene Blue (MB)”—the first phenothiazinium dye—whose synthesis protocol was patented by Heinrich Caro in the 1870s (Caro, 1878).

**Figure 2:**
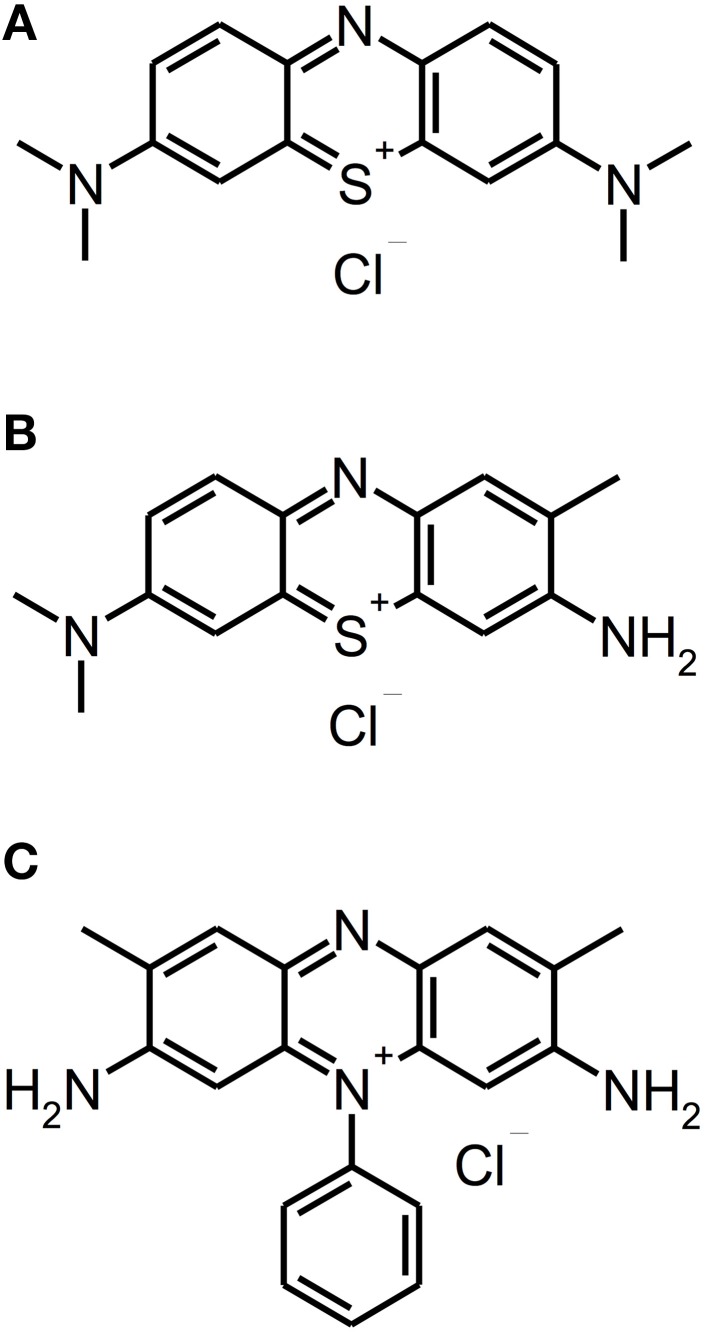
**Phenothiazinium derivatives**. Chemical structures of phenothiazinium derivatives: **(A)** Methylene Blue. **(B)** Toluidine Blue. **(C)** Safranine O.

Besides other applications, e.g., anti-malarial drugs or antipsychotic agents, phenothiazinium derivatives have been employed as PS due to their strong absorption in the red spectral region (ca. 600–680 nm) (Felgenträger et al., 2013). Phenothiazinium dyes are single positively charged and composed of a three-ring π-system with attached auxochromic side groups, whereby their singlet oxygen quantum yield Φ_Δ_ is around 0.5, thus acting according to type I and type II mechanisms (Wilkinson et al., 1993). Examples for phenothiazinium dyes, which have been tested for inactivation of biofilms *in vitro*, are foresaid MB [3,7-bis(dimethylamino)-phenothiazin-5-ium chloride] or Toluidine Blue [TBO; 3-amino-7-(dimethylamino)-2-methyl-phenothiazin-5-ium chloride] (see Figure 2).

Using MB, Fontana et al. investigated its effect on *ex vivo* polymicrobial biofilms cultivated from dental plaque samples taken from patients with chronic periodontitis. After a cultivation period of 7 days, the biofilms were incubated with MB in concentrations of 25 or 50 μg/ml for 5 min and illuminated with a diode laser (1 W; 665 nm) for another 5 min, whereby a light dose of 30 J/cm^2^ was applied, which resulted in a maximal inactivation rate of 32 % CFU only. The authors concluded that this low susceptibility of complex dental biofilms toward aPDT may be overcome by novel delivery and targeting approaches (Fontana et al., 2009).

For testing the efficacy of MB on yeast biofilms, Rossoni et al. (2014) cultured biofilms of *Candida albicans* serotype A and B strains for 48 h. After that period, biofilms were incubated with MB at a concentration of 300 μ M for 5 min and subsequently irradiated with a gallium-aluminum-arsenide (GaAlAs) laser (35 mW; 660 nm) for 285 s, applying a light dose of 26.3 J/cm^2^. This resulted in reductions of less than 1 log_10_ (64%) for serotype A biofilms, whereas for serotype B biofilms there was a reduction of more than 2 log_10_. The authors explained these distinct inactivation rates of *Candida albicans* serotype A and B biofilms by the differences in cell wall mannan structure between both serotypes. Furthermore, this difference in sensitivity to aPDT may be due to ultrastructural differences in EPS composition (Rossoni et al., 2014). However, in a comment to this study Mariusz Grinholc opposed that (i) only single reference strains of both serotypes and (ii) only one PS were tested, wherefore it may not be possible to conclude a general serotype-dependent sensitivity toward aPDT in cells of *Candida albicans* (Grinholc, 2014).

Employing TBO as a PS, the Brazilian group of Iriana C. J. Zanin conducted a number of studies (Zanin et al., 2005, 2006; Teixeira et al., 2012): In 2005 they examined its antimicrobial effect in combination with either a helium/neon (HeNe) gas laser (32 mW; 632.8 nm) or a light-emitting diode (LED; 32 mW; 620–660 nm with a maximum at 638.8 nm) on *Streptococcus mutans* biofilms grown on hydroxyapatite discs in a constant-depth film fermentor for 3, 7, or 10 days. After the respective culture period biofilms were incubated with TBO (100 mg/l) for 5 min and afterwards irradiated for 5, 15, or 30 min with HeNe laser or LED light (light doses of 49, 147, or 294 J/cm^2^). This resulted in reductions in viability of CFU of 2 to 5 log_10_ steps depending on biofilm age, light source and irradiation period (Zanin et al., 2005). One year later, they tested the effect of TBO and LED light on monospecies biofilms of *Streptococcus mutans*, *Streptococcus sobrinus*, and *Streptococcus sanguinis*. Here, biofilms were cultivated for 5 days on enamel slabs. After incubation with TBO (100 mg/l) for 5 min, biofilms were exposed to LED light (32 mW; 620–660 nm, maximum: 638.8 nm) for 7 min (light dose: 85.7 J/cm^2^). Results showed inactivation rates of approximately 1 log_10_ step for *Streptococcus mutans* and *Streptococcus sobrinus* biofilms and more than 3 log_10_ for *Streptococcus sanguinis* biofilms. The authors tried to explain these varying results by the ability of mutans streptococci like *Streptococcus mutans* and *Streptococcus sobrinus* to produce EPS to a greater extent than *Streptococcus sanguinis*, which is not among mutans streptococci (Zanin et al., 2006). In 2012, the same group published a combined *in vitro* and *in situ* report on the photodynamic effect of TBO: (i) biofilms of *Streptococcus mutans* were grown on hydroxylapatite discs for 5 days, (ii) volunteers wore intraoral devices with enamel slabs for 7 days under cariogenic challenge by dropping a sucrose solution 8 times per day. After the respective culture period biofilms were incubated with TBO (100 mg/l) for 5 min and irradiated with a LED light source (40 mW, 620–660 nm, maximum: 638.8 nm) for 15 min (light dose 55 J/cm^2^). For *in vitro* biofilms CFU were reduced by more than 5 log_10_ steps after aPDT, whereas for *in situ* biofilms reduction was less than 1 log_10_ step, when the numbers of CFU of *Streptococcus mutans* and total streptococci were examined. These distinct results were explained by the authors due to the thickness of the referring biofilms with *in situ* biofilms (1000 μm) being approximately tenfold as thick as *in vitro* biofilms (100 μm); therefore, they concluded that this problem could be solved e.g., by developing a PS being able to penetrate through the EPS (Teixeira et al., 2012).

As MB and TBO are designated to be potential efflux pump substrates in a variety of microbial species (Tegos and Hamblin, 2006; Prates et al., 2011) and due to the enhancing effect of efflux pump inhibitors on photodynamic inactivation of planktonic cells of Gram positives (Tegos et al., 2008), Kishen et al. (2010) tested the potentiating effect of an efflux pump inhibitor (verapamil hydrochloride) on aPDT with MB against *Enterococcus faecalis* monospecies biofilms. Hereby, 4 days old biofilms were incubated with MB or anionic RB (see below, Chapter Eosin Y, Erythrosine, Rose Bengal) as a control PS at concentrations of 100 μ M for 15 min in the dark and subsequently irradiated with a noncoherent light source with 30 nm bandpass filters (300–600 mW; MB: 660 ± 15 nm; RB: 540 ± 15 nm), employing light doses from 10 to 40 J/cm^2^. For MB, there was a killing efficacy of more than 5 log_10_ steps, which could even be enhanced slightly by an additional incubation with the efflux pump inhibitor. Employing RB exhibited only marginally worse results (≈5 log_10_). However, CLSM analysis showed that aPDT with these PS had different effects on the *Enterococcus faecalis* biofilms: While aPDT with RB produced no substantial destruction of biofilm structure, aPDT with MB resulted in damage of biofilm structure to a greater extent (Kishen et al., 2010).

In a study investigating the efficacy of two commercially available systems for aPDT, Meire et al. (2012) treated 24 h old biofilms of *Enterococcus faecalis* either with PAD™ (Dentofex, Inverkeithing, UK) or HELBO® (HELBO® Photodynamic Systems, Bredent medical, Senden, Germany) according to the recommendations of the manufacturers. For PAD™, biofilms were incubated for 2 min with TBO at a concentration of 12.7 mg/l and afterwards illuminated for 150 s with a soft diode laser (100 mW) emitting at 635 nm. For HELBO®, incubation of the *Enterococcus faecalis* biofilms was with MB (10 mg/ml) for 3 min with a subsequent irradiation for 2 min with a soft laser (HELBO® Theralite Laser) obtaining a wavelength of 660 nm and an output-power of 75 mW. Results showed a 2 log_10_ step reduction for HELBO®, whereas PAD™ exhibited only an inactivation rate of less than 1 log_10_ step. When in addition the antibacterial efficacy of irradiation with Er:YAG (2940 nm; 50 or 100 mJ; 15 Hz; 40 s) and Nd:YAG (1064 nm; 2 W; 15 Hz; 40 s) lasers was tested, there was a 4 log_10_ inactivation rate for Er:YAG treatment using 100 mJ pulses, whereas Er:YAG using 50 mJ pulses and Nd:YAG had no effect at all (<1 log_10_). In contrast, treatment with NaOCl (2.5%; 1, 5, 10, or 30 min) showed reductions of more than 6 log_10_ regardless which immersion periods were used. However, a prolonged action of NaOCl beyond the respective treatment periods cannot be ruled out, since no reagent was used for stopping its antimicrobial effect after the respective immersion period (Meire et al., 2012).

However, in addition to the above-mentioned batch-culture studies, phenothiazinium derivatives have also been examined in more applied models: Zand et al. (2014) used extracted human maxillary and mandibulary incisors, decoronated them and instrumented their root canals up to #60 K-file. After an autoclavation process *Enterococcus faecalis* biofilms were formed in these root canals for 4, 6 or 8 weeks. Subsequently, canals were incubated with TBO (25 μg/ml) for 5 min and irradiated with a 625 nm diode laser (100 mW; light dose 214.28 J/cm^2^) twice for 2.5 min intermitted by a 2.5 min break, applying a total of 2 × 15 J, which led to a complete elimination of *Enterococcus faecalis* below detection limit of the CFU assay (Zand et al., 2014).

In contrast, Fimple et al. (2008) investigated the photodynamic effects of MB on multispecies biofilms comprising *Actinomyces israelii*, *Fusobacterium nucleatum*, *Prevotella intermedia*, and *Porphyromonas gingivalis* in infected root canals of extracted human teeth. Teeth were decoronated, instrumented up to an apical file size of 0.465, autoclaved and infected with the foresaid pathogens. After a culture period of 3 days canals were incubated with MB (25 μg/ml) for 10 min and irradiated by means of a diode laser (1 W; 665 nm) coupled to an optical fiber for 2.5 min followed by a 2.5 min break and a second light exposure of 2.5 min, applying a total light dose of 30 J/cm^2^. Results showed a CFU reduction by 80%. The authors concluded that aPDT with MB could be an effective adjunct to conventional endodontic treatment, when the parameters get optimized (Fimple et al., 2008).

Up to date, phenothiazinium derivatives have to be regarded as the most studied PS in respect of inactivation of biofilms. Moreover, recently Voos et al. (2014) examined Safranine O (3,7-diamino-2,8-dimethyl-5-phenyl-phenazinium chloride), a structure analog to classical phenothiazinium dyes, where a nitrogen atom replaces the sulfur atom, which centers the delocalized electron system, for aPDT against biofilms. In this study, *ex vivo* biofilms were cultured from subgingival plaque samples for 24 or 72 h. After the respective incubation period the biofilms were incubated with Safranine O (10 μ M) for 15 min followed by irradiation with a diode laser (0.5 W; 532 nm) for 40 or 100 s, applying light doses of 20 or 50 J/cm^2^, respectively. In 24 h old biofilms there was a reduction of 2 log_10_ steps with a light dose of 20 J/cm^2^ and 3 log_10_ steps with 50 J/cm^2^, whereas treatment with CHX (0.2%; 3 min) generated a reduction by 1 log_10_ step only. In contrast, in 72 h old biofilms neither aPDT nor treatment with CHX led to any antibacterial effect (Voos et al., 2014). (Please find a summary of all studies described in this section in Table 1).

**Table 1:** **General characteristics of studies examining phenothiazinium derivatives**.

**References**	**Bacteria (parameters of biofilm-cultivation)**	**PS**	**Concentration**	**Incubation period**	**Light source (output power/intensity; wavelength)**	**Irradiation period**	**Light dose; applied energy**	**Overall efficacy (reduction of CFU)**	3 log_**10**_? ^*^
Fontana et al., 2009	*Ex vivo* (dental plaque samples; 7 days)	MB	25 μg/ml	5 min	Diode laser (1 W; 665 nm)	5 min	30 J/cm^2^	8%	No
			50 μg/ml					32%	
Rossoni et al., 2014	*C. albicans* (monospecies; 48 h)	MB	300 μ M	5 min	GaAlAs laser (35 mW; 660 nm)	285 s	26.3 J/cm^2^	Serotype A: ≥2 log_10_	No
								Serotype B: ≤1 log_10_	No
Zanin et al., 2005	*S. mutans* (monospecies; 3, 7, or 10 days)	TBO	100 mg/l	5 min	HeNe gas laser (32 mW; 632.8 nm) or LED (32 mW; 620–660 nm, maximum: 638.8 nm)	5, 10, or 15 min	49, 147, or 294 J/cm^2^	2–5 log_10_	Yes
Zanin et al., 2006	*S. mutans*, *S. sobrinus*, *S. sanguinis* (monospecies; 5 days)	TBO	100 mg/l	5 min	LED (32 mW, 620–660 nm, maximum: 638.8 nm)	7 min	85.7 J/cm^2^	*S. m.*, *S. sobr.*: ≈ 1 log_10_	No
								**- - - - - - - - - - - - - - - -**	**- - - - - -**
								*S. sang.*: ≥3 log_10_	Yes
Teixeira et al., 2012	*S. mutans* (monospecies; 5 days)	TBO	100 mg/l	5 min	LED (40 mW, 620–660 nm, maximum: 638.8 nm)	15 min	55 J/cm^2^	≥5 log_10_	Yes
	**- - - - - - - - - - -**							**- - - - - - - - - - - - - - - -**	**- - - - - -**
	*In situ* (7 days cariogenic challenge; evaluation of *S. mutans* and total streptococci)							< 1 log_10_	No
Kishen et al., 2010	*E. faecalis* (monospecies; 4 days)	MB	100 μ M	15 min	Noncoherent light source with 30 nm bandpass filters (300–600 mW; MB: 660 ± 15 nm; RB: 540 ± 15 nm)	n/s	10–40 J/cm^2^	≥5 log_10_	Yes
		**- - - - - - - - - -**	**- - - - - - - - - -**	**- - - - - - - - - -**		**- - - - - - - - -**	**- - - - - -**	**- - - - - - - - - - - - - - - -**	**- - - - - -**
		RB	100 μ M	15 min		n/s	10–40 J/cm^2^	≈ 5 log_10_	Yes
Meire et al., 2012	*E. faecalis* (monospecies; 24 h)	TBO (PAD™)	12.7 mg/l	2 min	Soft diode laser (100 mW; 635 nm)	2.5 min	n/s	<1 log_10_	No
		**- - - - - - - - - -**	** - - - - - - - - - -**	**- - - - - - - - - -**	**- - - - - - - - - - - - - - - - - - -**	**- - - - - - - - - -**	**- - - - - - - - - -**	**- - - - - - - - - -**	**- - - - - -**
		MB (HELBO®)	10 mg/ml	3 min	Soft laser (75 mW; 660 nm)	2 min	n/s	≈ 2 log_10_	No
		**- - - - - - - - - -**	**- - - - - - - - - -**	**- - - - - - - - - -**	**- - - - - - - - - - - - - - - - - -**	**- - - - - - - - - -**	**- - - - - - - - - -**	**- - - - - - - - - -**	**- - - - - -**
		NaOCl	2.5%	1, 5, 10, or 30 min				≥6 log_10_	Yes
		**- - - - - - - - - -**	**- - - - - - - - - -**	**- - - - - - - - - -**	**- - - - - - - - - - - - - - - - - - - -**	**- - - - - - - - - -**	**- - - - - - - -**	**- - - - - - - - - -**	**- - - - - -**
					Er:YAG laser (2940 nm; 15 Hz)	40 s	50 mJ	<1 log_10_	No
							100 mJ	≥4 log_10_	Yes
					**- - - - - - - - - - - - - - - - - -**	**- - - - - - - - - -**	**- - - - - - - - - -**	**- - - - - - - - - -**	**- - - - - -**
					Nd:YAG laser (1064 nm; 15 Hz)	40 s	2 W	<1 log_10_	No
Zand et al., 2014	*E. faecalis* (monospecies; artificially infected root canals; 4, 6, or 8 weeks)	TBO	25 μg/ml	5 min	Diode laser (100 mW; 625 nm)	2 × 2.5 min (intermitted by 2.5 min break)	214.28 J/cm^2^ 30 J	Total elimination (below detection limit of CFU-assay)	Yes
Fimple et al., 2008	*A. israelii*, *F. nucleatum*, *P. gingivalis*, *P. intermedia* (polyspecies; artificially infected root canals; 3 days)	MB	25 μg/ml	10 min	Diode laser (1 W; 665 nm) coupled to an optical fiber	2 × 2.5 min (intermitted by 2.5 min break)	30 J/cm^2^	80%	No
Voos et al., 2014	*Ex vivo* (subgingival plaque samples; 24 or 72 h)	Safranine O	10 μ M	15 min	Diode laser (0.5 W; 532 nm)	40 s	20 J/cm^2^	24 h: 2 log_10_	No
								72 h: -	No
						100 s	50 J/cm^2^	24 h: 3 log_10_	Yes
								72 h: -	No

* According to the ASM it is necessary to prove a reduction of 3 log_10_ steps of CFU for being able to use the terms “antimicrobial” or “antibacterial.”

### Porphyrins, chlorins, and phthalocyanines

**Figure 3:**
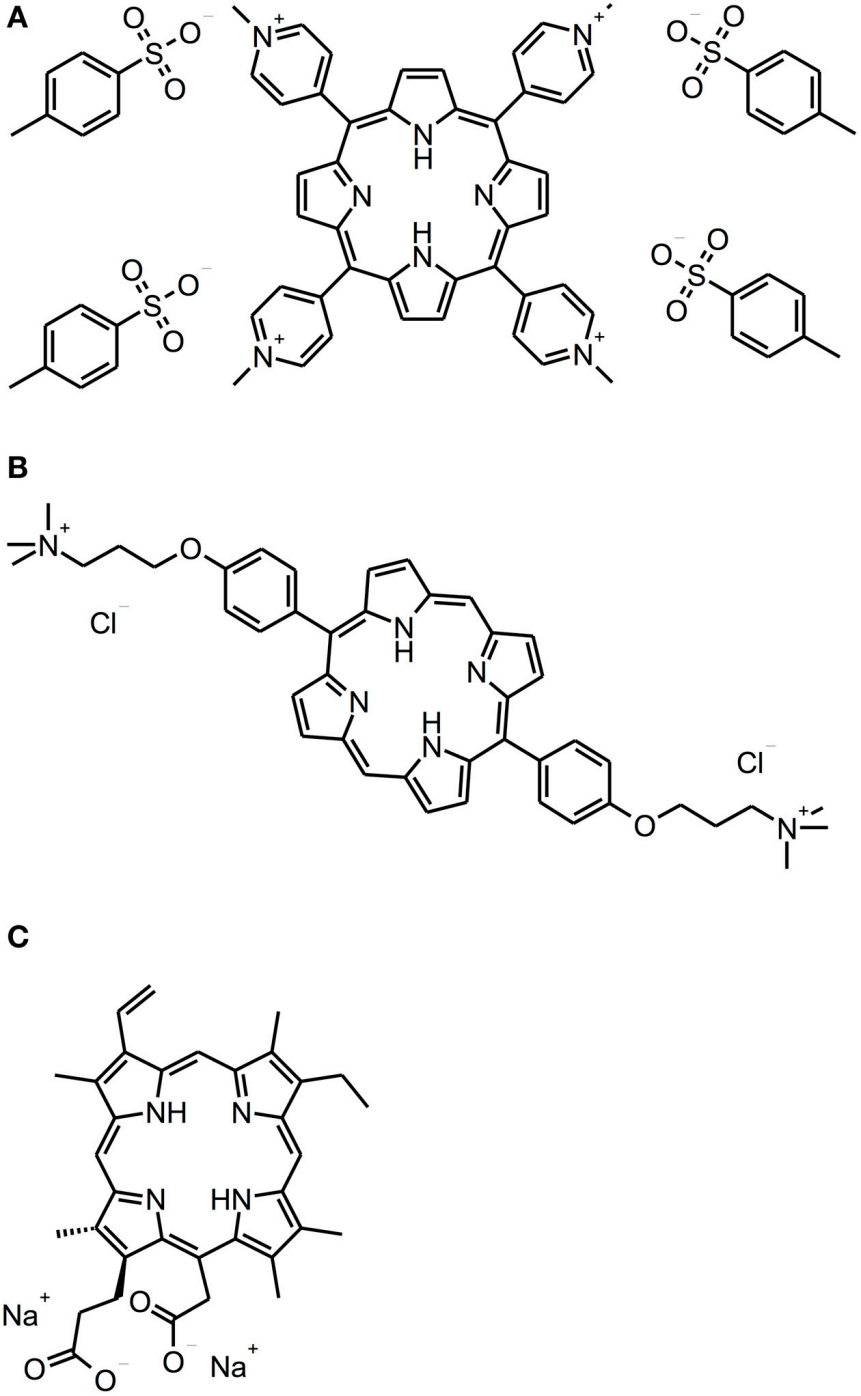
**Porphyrin and chlorin derivatives**. Chemical structures of porphyrin and chlorin derivatives: **(A)** TMPyP. **(B)** XF-73. **(C)** Photodithazine®.

Porphyrins, chlorins, and phthalocyanines are structurally comparable heterocyclic macrocycles, whereby porphyrins and phthalocyanines are composed of four pyrrole subunits, while chlorins comprise three pyrrole cycles and one pyrroline (see Figure 3). Many of these compounds are naturally occurring. For example, one of the widest known porphyrins is heme, the pigment in erythrocytes, on which the red color of blood is based. Likewise, some bacterial species form porphyrins in their metabolism.

Oral bacteria that are able to synthetize for example black-pigmented species like *Porphyromonas gingivalis* and *Prevotella* spp. (Soukos et al., 2005; Lennon et al., 2006) or *Aggregatibacter actinomycetemcomitans* (Cieplik et al., 2013a). Consequently, it has been shown *in vitro* that these bacterial species can be inactivated by irradiation with light only, whereby it is a commonly accepted hypothesis that endogenous porphyrins among other substances may act as PS and lead to a lethal auto-photosensitization process (König et al., 2000; Soukos et al., 2005; Lennon et al., 2006; Cieplik et al., 2013a). Porphyrins and chlorins have an intense absorption maximum at approximately 405 nm, known as Soret band, and smaller peaks at wavelengths longer than 500 nm (Q bands) (Gouterman, 1961); their singlet oxygen quantum yields are in a range between 0.5 and 0.8 (Fernandez et al., 1997), thus acting predominantly according to type II mechanism.

There are some studies evaluating the efficacy of cationic PS being based on porphyrin or chlorin structures concerning the inactivation of biofilms. With respect to porphyrins, TMPyP [5,10,15,20-tetrakis(1-methyl-4-pyridinium)-porphyrin tetra-(p-toluenesulfonate]—a fourfold positively charged derivative—has to be regarded as the most commonly used PS (Di Poto et al., 2009; Collins et al., 2010; Maisch et al., 2012; Cieplik et al., 2013b; Eichner et al., 2013; Gonzales et al., 2013). Di Poto et al. (2009) cultured biofilms from three distinct strains of *Staphylococcus aureus* for 24 h, incubated them with TMPyP at a concentration of 10 μ M for 15 min and irradiated them with increasing doses of white light (150–200 J/cm^2^) isolated from the emission of a tungsten lamp (166 mW/cm^2^; 400–800 nm), whereby this treatment exhibited a rate of bacteria killing of 1 to 2 log_10_ steps at the highest light dose depending on the strain, which was tested. Hereby, CLSM analysis showed the presence of dead cells throughout the biofilm implying that there was no hindrance for the PS to diffuse into the biofilms; consequently, the authors suggested that the reduced susceptibility of biofilm bacteria compared to that, which was observed when planktonic bacteria were treated (≥6 log_10_), may be due to differences in cell wall composition, growth rate or EPS-components hindering uptake of the PS through the cell walls. Furthermore, aPDT displayed a second anti-biofilm mechanism in this study leading to detachment of parts of the biofilm and therefore to a disruption of biofilm architecture (Di Poto et al., 2009).

Collins et al. (2010) tested the effect of aPDT with TMPyP on *Pseudomonas aeruginosa* biofilms, which were cultured for 24 h from a wild type or a mutant strain, respectively. After that period, 225 μ M TMPyP was applied, immediately followed by illumination with a mercury vapor lamp fitted with a colored glass filter blocking wavelengths shorter than 400 nm (100 W; ≥400 nm) for 10 min (220–240 J/cm^2^), which resulted in a pronounced inactivation rate of about 4 log_10_ steps for both strains. In addition, CLSM analysis revealed that aPDT resulted in substantial disruption of wild-type biofilms, whereby TMPyP without irradiation did not have that effect (Collins et al., 2010).

In contrast, when our group used TMPyP for aPDT against monospecies biofilms of *Enterococcus faecalis* (as a control PS for evaluating the antibacterial efficacy of SAPYR, which will be discussed below) we found no reduction of CFU at all. In this study, after a cultivation period of 72 h, biofilms were incubated with TMPyP (100 μ M) for 60 min and irradiated for 2 min with a LED light-curing unit for dental resins (1360 mW/cm^2^; 460 ± 20 nm), whereby the intensity of light reaching the biofilms was 600 mW/cm^2^. This inefficacy may be due to the large molecular structure of TMPyP, which may hinder the penetration of this PS throughout the EPS due to steric reasons. Furthermore, drug diffusion may also be delayed due to strong electrostatic interactions between fourfold positively charged TMPyP and negatively charged EPS-molecules. The emission of the LED light-curing unit was also not ideal for excitation of either SAPYR or TMPyP; nevertheless, this type of light source was chosen, as it is widely applied in dental practice (Cieplik et al., 2013b).

Pereira Gonzales et al. (2013) compared the antimicrobial efficacy of XF-73 [5,15-bis-[4-(3-trimethylammoniopropyloxy)-phenyl]-porphyrin chloride]—a porphyrin derivative with two positive charges only—to that of TMPyP for inactivation of *Candida albicans* biofilms. These biofilms were cultured for 24 h, incubated with increasing concentrations of PS for 4 h and illuminated with an incoherent light source (13.4 mW/cm^2^; 418 ± 20 nm) for 60 min, whereby a light dose of 48.2 J/cm^2^ was applied. Results showed CFU reductions of more than 5 log_10_ for concentrations of 1.0 μ M of XF-73 at least, whereas for TMPyP concentrations of 50 μ M were needed at the minimum for obtaining equal antimicrobial results. This was explained due to stronger electrostatic interactions of TMPyP with the EPS compared to XF-73 leading to a lower degree of diffusion of the PS into the biofilm (Gonzales et al., 2013).

Referring to chlorins, Photodithazine® is a commercially available cationic chlorin-*e*6 derivative with two absorption maxima at around 400 nm (Soret band) and 660 nm (slightly smaller peak, Q-band), which has been evaluated in two studies conducted by the group of Ana C. Pavarina (Dovigo et al., 2013; Quishida et al., 2013): Quishida et al. formed multispecies biofilms from *Streptococcus mutans*, *Candida albicans* and *Candida galbrata* for 48 h; afterwards they were incubated with Photodithazine® at concentrations from 100 to 250 mg/l for 20 min and exposed to red light from a LED light source (71 mW/cm^2^; 660 nm) for 9 min (light dose 37.5 J/cm^2^). At a PS-concentration of 200 mg/ml aPDT-treatment showed reductions of 1 or 2 log_10_ steps for *Candida* spp. or *Streptococcus mutans*, respectively (Quishida et al., 2013). In another study from the same group, Dovigo et al. cultured monospecies biofilms of clinical isolates of *Candida* spp. according to the protocol outlined above. These were incubated with Photodithazine® (125 mg/l) for 20 min and afterwards illuminated with a LED device (25 mW/cm2; 660 ± 20 nm) applying a light dose of 37.5 J/cm^2^. Results showed reductions of approximately 1 log_10_ step (Dovigo et al., 2013). However, in both of these studies Photodithazine® was excited by red light at its minor absorption peak at 660 nm without this point being discussed by the authors. Consequently, irradiation with blue light at 400 nm may lead to better results (Dovigo et al., 2013; Quishida et al., 2013).

In contrast to porphyrins and chlorins, phthalocyanines are highly hydrophobic, which compromises their water solubility (Ribeiro et al., 2013b). Therefore, methods for solubilization of phthalocyanines are mandatory for applying these compounds as PS for aPDT, whereby one option is their entrapment in nanoemulsions. Ribeiro et al. evaluated chloro-aluminum phthalocyanine (ClAlPc) encapsulated in cationic and anionic nanoemulsions (final concentration of ClAlPc in nanoemulsions: 31.8 μ M) in two studies regarding its aPDT-efficacy on biofilms of *Candida albicans* (Ribeiro et al., 2013a,b). However, in these studies the antimicrobial efficacy was only examined by detecting metabolic cell activity with XTT reduction assay. Consequently, this data is not comparable to CFU-assay data, which is mandatory, since—according to the ASM—any novel approach has to accomplish a CFU reduction rate of more than 3 log_10_ in order to use the terms “antibacterial” or “antimicrobial” (Table 2).

**Table 2:** **General characteristics of studies examining porphyrin and chlorin derivatives**.

**References**	**Bacteria (parameters of biofilm-cultivation)**	**PS**	**Concentration**	**Incubation period**	**Light source (output power/intensity; wavelength)**	**Irradiation period**	**Light dose; applied energy**	**Overall efficacy (reduction of CFU)**	3 log_**10**_? ^*^
Di Poto et al., 2009	*S. aureus* (monospecies; 24 h)	TMPyP	10 μ M	15 min	Tungsten lamp (166 mW/cm^2^; 400–800 nm)	n/s	150–200 J/cm^2^	1 to 2 log_10_	No
Collins et al., 2010	*P. aeruginosa* (monospecies; 24 h)	TMPyP	225 μ M	0 min	Mercury vapor lamp (100 W; ≥ 400 nm)	10 min	220–240 J/cm^2^	4 log_10_	Yes
Cieplik et al., 2013b	*E. faecalis* (monospecies; 72 h)	TMPyP	100 μ M	60 min	LED light-curing unit (1360 mW/cm^2^; at sample level: 600 mW/cm^2^; 460 ± 20 nm)	120 s	Total absorbed energy: 80 J	No effect	No
	**- - - - - - - - - - - - - -**	**- - - - - - - - -**	**- - - - - - - - -**	**- - - - - - - - -**		**- - - - - - - -**	**- - - - - - - - -**	**- - - - - - - -**	**- - - - - -**
	*E. faecalis* (monospecies; 72 h)	SAPYR	100 μ M	60 min		120 s	total absorbed energy: 12.5 J	≥5 log_10_	Yes
	*E. faecalis* (polyspecies; 72 h)							≥5 log_10_	Yes
	*A. naeslundii* (monospecies; 72 h)							≥2 log_10_	Yes
	*A. naeslundii* (polyspecies; 72 h)							≥4 log_10_	Yes
Gonzales et al., 2013	*C. albicans* (monospecies; 24 h)	TMPyP	0.1–50 μ M	4 h	Incoherent light source (at sample level: 13.4 mW/cm^2^; 418 ± 20 nm)	60 min	48.2 J/cm^2^	50 μ M: ≥5 log_10_	Yes
		**- - - - - - - - -**	**- - - - - - - - -**	**- - - - - - - - -**		**- - - - - - - - -**	**- - - - - - - - -**	**- - - - - - - -**	**- - - - - -**
		XF-73	0.1–10 μ M	4 h		60 min	48.2 J/cm^2^	1 μ M: ≥5 log_10_	Yes
Quishida et al., 2013	*S. mutans*, *C. albicans*, *C. galbrata* (polyspecies; 48 h)	Photodithazine®	100–250 mg/l	20 min	LED (71 mW/cm^2^; 660 nm)	9 min	37.5 J/cm^2^	*C.* spp.: 1 log_10_	No
								*S. m.*: 2 log_10_ (for 175 or 200 mg/ml)	No
Dovigo et al., 2013	Clinical isolates of *Candida* spp. (monospecies; 48 h)	Photodithazine®	125 mg/l	20 min	LED (25 mW/cm^2^; 660 ± 20 nm)	n/s	37.5 J/cm^2^	≈ 1 log_10_	No

* According to the ASM it is necessary to prove a reduction of 3 log_10_ steps of CFU for being able to use the terms “antimicrobial” or “antibacterial.”

### Eosin Y, Erythrosine, rose bengal

**Figure 4:**
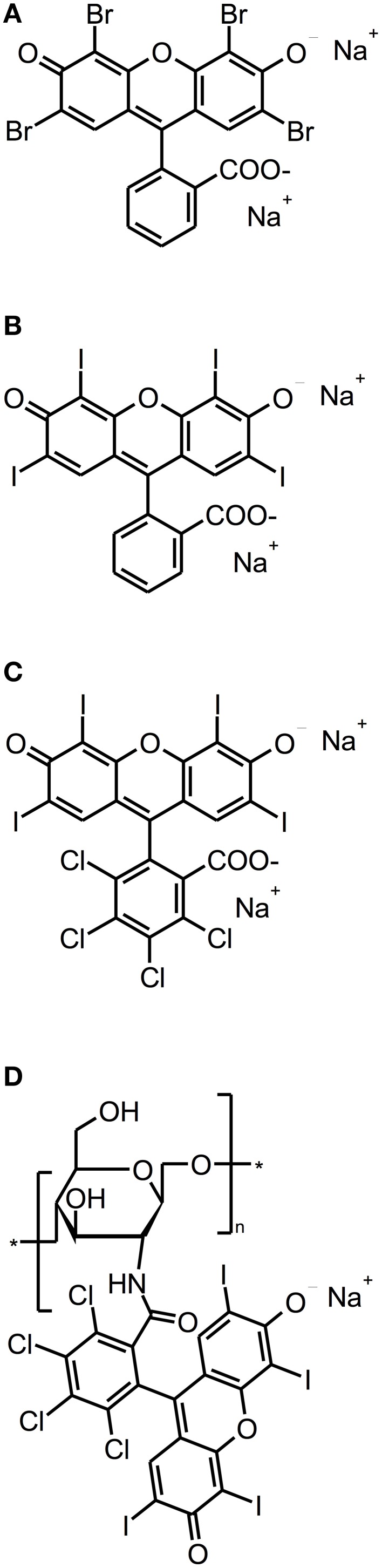
**Fluorescein derivatives**. Chemical structures of fluorescein derivatives: **(A)** Eosin Y. **(B)** Erythrosine. **(C)** Rose Bengal. **(D)** Chitosan-conjugated Rose Bengal.

Eosin Y, Erythrosine and Rose Bengal (RB) are anionic xanthene dyes derived from fluorescein. Hereby, Eosin Y and Erythrosine are red dyes resulting from the action of either bromine or iodine on fluorescein, whereas RB is a pink dye eventuating from a tetrachlorination of Erythrosine (see Figure 4). All these dyes show intense absorption bands in the green wavelength range (480–550 nm) (DeRosa and Crutchley, 2002); their singlet oxygen quantum yields are between 0.6 and 0.8, therefore mainly acting according to type II mechanism (Wilkinson et al., 1993).

Employing Erythrosine (ERY; 2,4,5,7-tetraiodofluorescein) as a PS for aPDT, Wood et al. (2006) studied its effect on 200 μm thick biofilms of *Streptococcus mutans*, which were grown for up to 288 h in a constant-depth film fermentor, and compared it to MB and Photofrin, a Porphyrin derivative. The biofilms were incubated with 22 μ M ERY, MB, or Photofrin for 15 min and exposed to white light from a tungsten filament lamp (ERY: 22.7 mW/cm^2^ in the wavelength range 500–550 nm; MB, Photofrin: 22.5 mW/cm^2^ at 600–650 nm) for 15 min. Hereby, ERY-mediated aPDT resulted in a reduction of about 3 log_10_ steps for 288 h old biofilms, whereas aPDT with MB and Photofrin showed only 2.6 or 1.1 log_10_ steps inactivation, respectively. Contrary to expectations, 48 h old biofilms were less susceptible to aPDT, regardless which PS was used (ERY: 2.2 log_10_; MB: 1.5 log_10_; Photofrin: 0.5 log_10_). The authors explained this as young biofilms may be more metabolically active, thus having more effective repair systems. Moreover, older biofilms may contain voids and channels through the EPS, allowing greater amount of penetration of the respective PS (Wood et al., 2006). However, negatively charged ERY being the most effective PS in this study is quite contradictory to the above-mentioned general opinion, how a “perfect” PS should be composed, as the positive charge of a PS appears to promote an electrostatic interaction with negatively charged sites at the outer membrane of bacterial cells (Maisch et al., 2004). In contrast, anionic fluorescein derivatives like ERY or RB may be efficient though due to their high lipophilicity. In another study of the same group, Metcalf et al. (2006) investigated the effect of light-fractionation on aPDT with ERY on *Streptococcus mutans* biofilms formed under equal experimental parameters. ERY was used at 22 μ M with a 15 min incubation period, too. Biofilms were illuminated with the same tungsten filament lamp (22.7 mW/cm^2^; 500–550 nm), whereby a light dose of 6.75 J/cm^2^ was applied for every 5 min of irradiation. Here, it was shown that aPDT with ERY can be potentiated by light fractionation: *Streptococcus mutans* could be inactivated by about 2 log_10_ steps, when ERY was irradiated continuously for 5 min, whereas rates of 3 log_10_ and 3.7 log_10_ could be achieved, when there were either 5 × 1 min light pulses with 5 min recovery periods or 10 × 30 s light pulses with 2 min recovery breaks between pulses. This was explained by the authors due to replenishment of target molecules such as oxygen for the PS during the dark periods (Metcalf et al., 2006).

In order to compare the photodynamic efficacy of ERY and RB (4,5,6,7-tetrachloro-2′,4′,5′,7′-tetraiodofluorescein), Pereira et al. (2013) cultured biofilms of *Streptococcus mutans* and *Streptococcus sanguinis* for 48 h and incubated them with either with ERY or RB at concentrations of 5 μ M for 5 min followed by irradiation with a blue LED (200 mW; 455 ± 20 nm) for 180 s (light dose: 95 J/cm^2^; applied energy: 36 J). This treatment exhibited small effects of less than 1 log_10_ for both species regardless of whether ERY or RB was used. However, it has to be considered that blue light from a 455 nm LED may not be optimal for excitation of ERY and RB with green light being more appropriate (Pereira et al., 2013). In a study from the same group, Freire et al. (2013) compared RB with Eosin Y (2,4,5,7-tetrabromofluorescein) concerning their potency for inactivation of *Candida albicans* biofilms. After culturing these biofilms for 48 h, treatment was done with 200 μ M RB or Eosin Y for 5 min as a pre-irradiation period and subsequent illumination with a green LED (90 mW; 532±10 nm) for 180 s (light dose: 42.63 J/cm^2^; applied energy: 16.2 J), which resulted in inactivation rates of 0.22 and 0.45 log_10_ for RB and Eosin Y, respectively (Freire et al., 2013). In contrast to those results, Kishen et al. achieved a high killing efficacy of approximately 5 log_10_ steps, when using RB against *Enterococcus faecalis* monospecies biofilms, as mentioned above (see Chapter Phenothiazinium Derivatives) (Kishen et al., 2010).

However, as cationic PS are needed for good antimicrobial photodynamic activity, the group of Annie Shrestha and Anil Kishen synthesized a polycationic chitosan-conjugated Rose Bengal (CSRB) for mounting a positive charge on anionic RB (Shrestha and Kishen, 2012; Shrestha et al., 2012). This conjugate was evaluated against *Enterococcus faecalis* and *Pseudomonas aeruginosa* biofilms, whereby its efficacy was compared to RB and MB. Biofilms were cultured for 7 days, incubated for 15 min with the respective PS at concentrations of 0.3 mg/ml (CSRB) or 10 μ M (RB, MB) and irradiated by 540 nm (CSRB, RB) or 660 nm (MB) fibers. Applying a light dose of 40 J/cm^2^ resulted in inactivation rates of about 3 log_10_ steps for MB in both biofilms, while for RB there were reductions of 3 log_10_ steps in *Pseudomonas aeruginosa* biofilms and 4 log_10_ steps in *Enterococcus faecalis* biofilms. In contrast, when using CSRB there was an inactivation of 9 log_10_ steps of *Pseudomonas aeruginosa* and 5 log_10_ steps of *Enterococcus faecalis*. The authors explained these strikingly better results of CSRB compared to anionic RB and cationic MB due to the greater ability of CSRB to adhere to bacterial cells and the synergistic antibacterial effects of chitosan and RB (Shrestha and Kishen, 2012) (Table 3).

**Table 3:** **General characteristics of studies examining fluorescein derivatives**.

**References**	**Bacteria (parameters of biofilm-cultivation)**	**PS**	**Concentration**	**Incubation period**	**Light source (output power/intensity; wavelength)**	**Irradiation period**	**Light dose; applied energy**	**Overall efficacy (reduction of CFU)**	3 log_**10**_? ^*^
Wood et al., 2006	*S. mutans* (monospecies; 200 μm; 48 to 288 h)	ERY	22 μ M	15 min	Tungsten filament lamp (ERY: 22.7 mW/cm^2^; 500–550 nm; MB, Photofrin: 22.5 mW/cm^2^; 600–650 nm)	15 min	n/s	48 h: 2.2 log_10_	No
								288 h: 3 log_10_	Yes
		**- - - - - - -**	**- - - - - - -**	**- - - - - - -**		**- - - - - - -**	**- - - - - - -**	**- - - - - - -**	**- - - - - - -**
		MB	22 μ M	15 min		15 min	n/s	48 h: 1.5 log_10_	No
								288 h: 2.6 log_10_	No
		**- - - - - - -**	**- - - - - - -**	**- - - - - - -**		**- - - - - - -**	**- - - - - - -**	**- - - - - - -**	**- - - - - - -**
		Photofrin	22 μ M	15 min		15 min	n/s	48 h: 0.5 log_10_	No
								288 h: 1.1 log_10_	No
Metcalf et al., 2006	*S. mutans* (monospecies; 200 μm)	ERY	22 μ M	15 min	Tungsten filament lamp (ERY: 22.7 mW/cm^2^; 500–550 nm)	5 min	6.75 J/cm^2^	2 log_10_	No
						**- - - - - - -**	**- - - - - - -**	**- - - - - - -**	**- - - - - - -**
						5 × 1 min (5 min breaks)	6.75 J/cm^2^	3 log_10_	Yes
						**- - - - - - -**	**- - - - - - -**	**- - - - - - -**	**- - - - - - -**
						10 × 30 s (2 min breaks)	6.75 J/cm^2^	3.7 log_10_	Yes
Pereira et al., 2013	*S. mutans*, *S. sanguinis* (monospecies; 48 h)	ERY	5 μ M	5 min	LED (200 mW; 455±20 nm)	180 s	95 J/cm^2^; 36 J	*S. m.*: 52%	No
								*S. s.*: 88%	No
		**- - - - - - -**	**- - - - - - -**	**- - - - - - -**		**- - - - - - -**	**- - - - - - -**	**- - - - - - -**	**- - - - - - -**
		RB	5 μ M	5 min		180 s	95 J/cm^2^; 36 J	*S. m.*: 62%	No
								*S. s.*: 95%	No
Freire et al., 2013	*C. albicans* (monospecies; 48 h)	RB	200 μ M	5 min	LED (90 mW; 532±10 nm)	180 s	42.63 J/cm^2^; 16.2 J	22%	No
		**- - - - - - -**	**- - - - - - -**	**- - - - - - -**		**- - - - - - -**	**- - - - - - -**	**- - - - - - -**	**- - - - - - -**
		Eosin Y	200 μ M	5 min		180 s	42.63 J/cm^2^; 16.2 J	45%	No
Kishen et al., 2010	*E. faecalis* (monospecies; 4 days)	RB	100 μ M	15 min	Noncoherent light source with 30 nm bandpass filters (300–600 mW; RB: 540±15 nm; MB: 660±15 nm)	n/s	10–40 J/cm^2^	≈ 5 log_10_	Yes
		**- - - - - - -**	**- - - - - - -**	**- - - - - - -**		**- - - - - - -**	**- - - - - - -**	**- - - - - - -**	**- - - - - - -**
		MB	100 μ M	15 min		n/s	10–40 J/cm^2^	≥ 5 log_10_	Yes
Shrestha and Kishen, 2012	*E. faecalis*, *P. aeruginosa* (monospecies; 7 days)	CSRB	0.3 mg/ml	15 min	Fibers (n/s; CSRB, RB: 540 nm; MB: 660 nm)	n/s	40 J/cm^2^	*E. f.*: ≈ 5 log_10_	Yes
								*P.a.*: ≈ 9 log_10_	Yes
		**- - - - - - -**	**- - - - - - -**	**- - - - - - -**		**- - - - - - -**	**- - - - - - -**	**- - - - - - -**	**- - - - - - -**
		RB	10 μ M	15 min		n/s	40 J/cm^2^	*E.f.*: ≈ 4 log_10_	Yes
								*P.a.*: ≈ 3 log_10_	Yes
		**- - - - - - -**	**- - - - - - -**	**- - - - - - -**		**- - - - - - -**	**- - - - - - -**	**- - - - - - -**	**- - - - - - -**
		MB	10 μ M	15 min		n/s	40 J/cm^2^	*E.f.*: ≈ 3 log_10_	Yes
								*P.a.*: ≈ 3 log_10_	Yes

* According to the ASM it is necessary to prove a reduction of 3 log_10_ steps of CFU for being able to use the terms “antimicrobial” or “antibacterial.”

### Curcumin, perinaphthenone and fullerene derivatives

**Figure 5:**
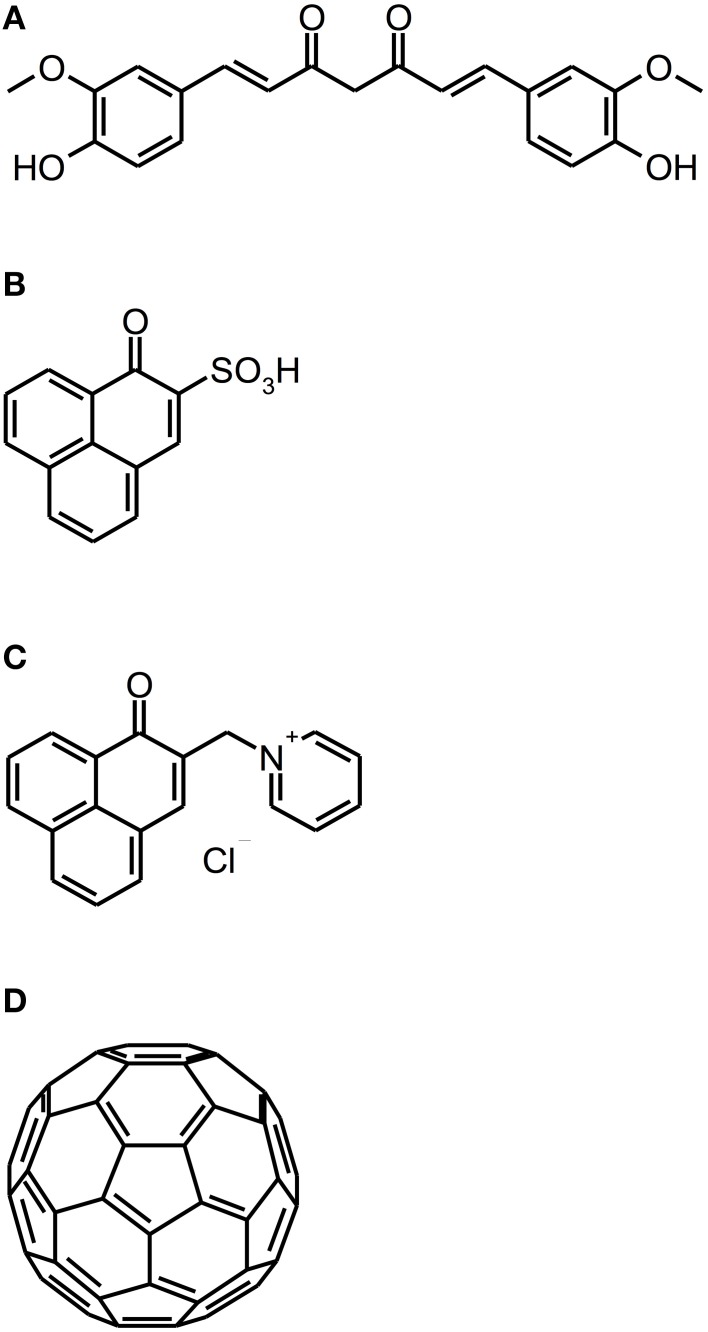
**Curcumin, perinaphthenone and fullerene derivatives**. Chemical structures of curcumin, perinaphthenone and fullerene derivatives: **(A)** Curcumin. **(B)** PNS. **(C)** SAPYR. **(D)** Fullerene C60.

Recently, new classes of PS have been introduced as PS for aPDT: Curcumins, perinaphthenone derivatives and fullerenes (see Figure 5).

Curcumin [1*E*,6*E*-1,7-bis(4-hydroxy-3-methoxyphenyl)-1,6-heptadiene-3,5-dione] is a naturally occurring intense-yellow dye, isolated from the rootstocks of the plant *Curcuma longa*, which has been used as medicine, spice and food-colorant for hundreds of years (Crivello and Bulut, 2005; Aggarwal et al., 2007). As its absorption spectrum is in the UV/blue wavelength range (300–500 nm with a maximum at 430 nm), it has been thought to be a feasible PS for aPDT (Araújo et al., 2012). However, curcumin is a nearly quantitative type I mechanism PS, whose photodynamic effect is due to hydrogen peroxide without generation of singlet oxygen (Dahl et al., 1989).

Araújo et al. (2014) evaluated the susceptibility of *Streptococcus mutans* and *Lactobacillus acidophilus*, either grown in multispecies biofilms or in artificial dentine carious lesions, toward aPDT with a curcumin-solution (consisting of 66.7% glucamin, 17.8% curcumin, 15.5% demethoxy and bisdemethoxy curcumin; concentration: 0.75 to 5 g/l). Biofilms were cultured for 7 days, exposed to the curcumin-solution for 5 min and irradiated for 5 min with blue light derived from a LED (central wavelength: 450 nm; light dose: 5.7 J/cm^2^). With curcumin-concentrations above 3 g/l, CFU were reduced by more than 3 log_10_ steps in the multispecies biofilms. However, when the dentine carious lesion was exposed to curcumin (5 g/l) with subsequent irradiation, there was only a small reduction of 69%; this reduced effect on bacteria located in demineralized dentine was explained by the authors due to decreased penetration depth of the PS, diminished binding to bacterial cells or attenuated penetration of light (Araújo et al., 2014).

In contrast to curcumins, perinaphthenones are only slight yellowish and exhibit singlet oxygen quantum yields close to unity, therefore acting nearly exclusively according to type II mechanism. Consequently, PNS (1H-phenalen-1-one-2-sulfonic acid), which was the first derivative of perinaphthenone being employed as a PS (originally described by Nonell et al., 1993), has been proposed as a universal standard for singlet oxygen studies (Oliveros et al., 1999). However, PNS is negatively charged, whereas positively charged PS are essential for good antimicrobial activity (Alves et al., 2009). Therefore, recently SAPYR [2-((4-pyridinyl)methyl)-1H-phenalen-1-one chloride] was introduced as the lead compound of a batch of positively charged derivatives based on a 7-perinaphthenone-structure, eliminating the drawback of PNS, but conserving the high singlet oxygen quantum yield of Φ_Δ_ 0.99 (Cieplik et al., 2013b; Späth et al., 2014). Our group evaluated SAPYR for its anti-biofilm properties against *Enterococcus faecalis* and *Actinomyces naeslundii* grown in monospecies or in polyspecies biofilms consisting of *Enterococcus faecalis*, *Actinomyces naeslundii*, and *Fusobacterium nucleatum* (Cieplik et al., 2013b). These biofilms were cultured for 72 h, afterwards incubated for 60 min with the respective PS at 100 μ M (TMPyP served as a reference PS) and irradiated for 120 s with a blue LED, usually applied for polymerization of dental resins (light intensity at sample-level: 600 mW/cm^2^). This light source was used, as it is widely applied in dental practice, although its emission spectrum shows only partial overlap with the absorption spectra of SAPYR and TMPyP. Nevertheless, aPDT with SAPYR resulted in CFU-reductions of more than 5 log_10_ for *Enterococcus faecalis* in both types of biofilm; *Actinomyces naeslundii* was inactivated by more than 2 log_10_ in monospecies and by more than 4 log_10_ in polyspecies biofilms. In contrast, as mentioned above (Section Porphyrins, Chlorins, and Phthalocyanines), TMPyP had no effect at all (Cieplik et al., 2013b).

As a completely distinct class of PS, fullerenes are ball-shaped cage molecules composed of carbon atoms, whereby fullerene C60 has to be regarded as the most studied compound (Sharma et al., 2011). Recently, the group of Michael R. Hamblin introduced several functionalized cationic fullerenes as PS for aPDT, which showed notable results *in vitro* against planktonic bacteria and yeasts (Mizuno et al., 2011). These compounds absorb extensively in the visible and UV wavelength range (Sharma et al., 2011) and react—in contrast to perinaphthenone derivatives—predominantly according to type I mechanism, thus producing mainly superoxide (Mizuno et al., 2011). However, until now fullerenes have not been tested for their antimicrobial photodynamic efficacy against biofilms (Table 4).

**Table 4:** **General characteristics of studies examining curcumin and perinaphthenone derivative**.

**References**	**Bacteria (parameters of biofilm-cultivation)**	**PS**	**Concentration**	**Incubation period**	**Light source (output power/intensity; wavelength)**	**Irradiation period**	**Light dose; applied energy**	**Overall efficacy (reduction of CFU)**	3 log_10_? ^*^
Araújo et al., 2012	*S. mutans*, *L. acidophilus* (polyspecies; 7 days)	Curcumin	0.75 to 5 g/l	5 min	LED (19 mW/cm^2^; central wavelength: 450 nm)	5 min	5.7 J/cm^2^	≥3 log_10_ (above 3 g/l)	Yes
	**- - - - - - - - - - - - - - - -**							**- - - - - - -**	**- - - - - - -**
	*S. mutans*, *L. acidophilus* (artificial dentine carious lesions; 7 days)							69% (at 5 g/l)	No
Cieplik et al., 2013b	*E. faecalis* (monospecies; 72 h)	SAPYR	100 μ M	60 min	LED light-curing unit (1360 mW/cm^2^; at sample level: 600 mW/cm^2^; 460 ± 20 nm)	120 s	Total absorbed energy: 12.5 J	≥5 log_10_	Yes
	*E. faecalis* (polyspecies; 72 h)							≥5 log_10_	Yes
	*A. naeslundii* (monospecies; 72 h)							≥2 log_10_	Yes
	*A. naeslundii* (polyspecies; 72 h)							≥4 log_10_	Yes
	**- - - - - - - - - - - - - -**	**- - - - - - -**	**- - - - - - -**	**- - - - - - -**		**- - - - - - -**	**- - - - - - -**	**- - - - - - -**	**- - - - - - -**
	*E. faecalis* (monospecies; 72 h)	TMPyP	100 μ M	60 min		120 s	Total absorbed energy: 80 J	No effect	No

* According to the ASM it is necessary to prove a reduction of 3 log_10_ steps of CFU for being able to use the terms “antimicrobial” or “antibacterial.”

## Synopsis

All above-mentioned studies have in common that they are difficult to compare with each other. In general, with respect to biofilm cultivation, different culture protocols with varying culture periods lead to different biofilms at distinct stages of development. This is a general problem, which has not been solved yet. While for testing a given antimicrobial against planktonic cultures, MIC (minimal inhibitory concentration), and MBC (minimal bactericidal concentration) assays are standardly used, protocols or assays for testing antimicrobials in biofilm models (e.g., the Calgary Biofilm Device described by Ceri et al., 1999) are not in widespread use. Consequently, the effect of a given PS on 24 h old biofilms described in one study can hardly be compared to the effect of the same PS on 4 weeks old biofilms described in another study. The ASM stated in 2010 that every new approach has to reach a 3 log_10_ step reduction rate of CFU before being able to use the terms “antimicrobial” or “antibacterial,” wherefore CFU assays are mandatory for testing PS for aPDT against biofilms. However, it has to be considered that it is a critical point to disaggregate bacteria from a biofilm before doing a classical CFU assay. Therefore, Live/Dead staining would be a suitable add-on tool for confirmation of results. Live/Dead staining cannot be used as the only evaluation method, though, as it only yields relative fluorescence counts without making an exact quantification possible.

Another point is that in most studies, which deal with inactivation of biofilms, the presence of an EPS has not been experimentally verified (e.g., by fluorescence microscopy), although—according to the general definition of a biofilm—this has to be regarded as a major criterium for using the term “biofim” (Donlan and Costerton, 2002). Consequently, in some cases the “biofilms” used may not be true biofilms, but rather attached bacteria without any EPS. Therefore, the robustness, reproducibility and relevance of the distinct biofilm assays, which were applied in the studies discussed above, remain debatable. In addition, it has to be considered that there is a remarkable difference between *in vitro* biofilms and biofilms formed *in vivo* on tooth or mucosal surfaces in the oral cavity. These clinical biofilms consist of hundreds of bacterial and fungal species obtaining a thickness of 1000 μm at least (Teixeira et al., 2012), whereas under laboratory conditions it is not able to mimic such complex biofilms. Nevertheless, from the studies implied in this review it can be concluded that there are not necessarily differences in inactivation rates between bacterial and fungal biofilms or between monospecies and polyspecies biofilms. In contrast, as shown by Teixeira et al. and also by our group, the thickness of an *in vitro* biofilm and its amount of EPS affect the efficacy of photodynamic inactivation, which may be due to the inability of a PS to diffuse throughout the entire biofilm (Teixeira et al., 2012; Cieplik et al., 2013b).

From a photophysical point of view, it is also problematic to compare the efficacy of two distinct PS on identically cultured biofilms, as absorption characteristics may vary for every PS. Therefore, the overlaps of two distinct PS with the emission of the same light source, thus the absorbed energies, can differ vastly even if the applied energies are equivalent. For example, this was shown by our group for TMPyP and SAPYR, where the total absorbed energies were 12.5 J for SAPYR vs. 80 J for TMPyP, while the applied energies were matching (Cieplik et al., 2013b). Giving the example of MB and SAPYR, different light sources have to be used, as MB is activated by red light, whereas SAPYR absorbs in the blue spectral range. Consequently, it is not reasonable to directly compare efficacy rates, which were revealed for a given PS in studies published by different groups.

Furthermore, for evaluation of the antimicrobial properties of a given PS it is essential to include appropriate control groups in the experimental protocol. Typically, in so-called negative controls, samples are treated either with PS only (PS+L-) or with light only (PS-L+) or remain completely untreated (PS-L). In contrast, positive controls mean that the efficacy of aPDT with a respective PS is compared to that of a drug, which has already demonstrated to exhibit pronounced antimicrobial properties; in dentistry, suitable drugs for positive control groups would be CHX or NaOCl. However, out of 22 studies reviewed in this paper, only one (Meire et al., 2012) used an antimicrobial drug (NaOCl) as a positive control. However, in this study no reagent was used for stopping the activity of NaOCl after the respective immersion period (Meire et al., 2012). It has to be considered that the treatment periods of suchlike antimicrobials are difficult to restrict when no reagents for stopping their antimicrobial effect are used, whereas in contrast the effect of aPDT is stopped immediately when the light is turned off, which reduces comparability between these approaches. For example, Hecker et al. showed that treatment with 1.0% and 3.0% NaOCl only had a biologically relevant effect (≥3 log_10_) on planktonic *Enterococcus faecalis*, which was grown in artificially infected bovine root canals, when the antimicrobial action of NaOCl was not arrested by adding sodium thiosulfate after the respective treatment period (30, 60, or 600 s). In contrast, when sodium thiosulfate was added, no biologically relevant effect (≥3 log_10_) could be observed. Therefore, the authors concluded that blocking the antimicrobial effect of a given antimicrobial after the required treatment period is essential for laboratory testing in order to avoid distorting results due to a prolonged action of the respective drug (Hecker et al., 2013).

All in all, the findings reviewed in the present paper imply that aPDT is an effective approach for inactivation of biofilms formed *in vitro*. Concerning the situation in the oral cavity, these *in vitro* results are promising for an application of aPDT as a supportive antimicrobial tool to combat biofilm-associated infections *in vivo*. Oral infections such as periodontal, endodontic or mucosal infections, and more recently periimplantitis represent—beside dermal infections—a superior field for application of aPDT due to their localized nature. Nevertheless, until now clinical oral application of aPDT is still in its infancy; clinical studies on aPDT were mainly conducted with phenothiazinium derivatives like MB and TBO as PS examining the potential of the photodynamic approach as a supportive tool for treatment of periodontitis, periimplantitis, or endodontic infection, whereby the results of these studies are quite conflicting (Gursoy et al., 2013). Moreover, using these PS, the patients' aesthetic may get compromised due to the strong blue color of these PS. For example, in periodontal application the oral soft tissues may get stained bluish for at least a few hours after treatment (Hayek et al., 2005; Sorkhdini et al., 2013). When used in endodontics, even persistent blue staining of dental structure via diffusion of the PS into dentinal tubules may get induced, by what a further decoloration step using appropriate chemical compounds may get necessary (Carvalho et al., 2011). Although there is current research on developing only slightly or even tooth-like colored PS, e.g., perinaphthenone derivatives like SAPYR (Cieplik et al., 2013b; Späth et al., 2014), further clinical research is needed—particularly on these new PS—for employing aPDT regularly in dental practice.

### Conflict of interest statement

The authors declare that the research was conducted in the absence of any commercial or financial relationships that could be construed as a potential conflict of interest.
